# The effect of activity-based financing on hospital length of stay for elderly patients suffering from heart diseases in Norway

**DOI:** 10.1186/1472-6963-13-172

**Published:** 2013-05-07

**Authors:** Jun Yin, Hilde Lurås, Terje P Hagen, Fredrik A Dahl

**Affiliations:** 1 Helse Sør-Øst Health Services Research Centre, Akershus University Hospital, Lørenskog, Norway; 2 Department of Health Management and Health Economics, University of Oslo, Oslo, Norway; 3 Institute of Clinical Medicine, University of Oslo, Oslo, Norway

**Keywords:** Activity-based financing, Length of hospital stay, Elderly, Ischemic heart diseases

## Abstract

**Background:**

Whether activity-based financing of hospitals creates incentives to treat more patients and to reduce the length of each hospital stay is an empirical question that needs investigation. This paper examines how the level of the activity-based component in the financing system of Norwegian hospitals influences the average length of hospital stays for elderly patients suffering from ischemic heart diseases. During the study period, the activity-based component changed several times due to political decisions at the national level.

**Methods:**

The repeated cross-section data were extracted from the Norwegian Patient Register in the period from 2000 to 2007, and included patients with angina pectoris, congestive heart failure, and myocardial infarction. Data were analysed with a log-linear regression model at the individual level.

**Results:**

The results show a significant, negative association between the level of activity-based financing and length of hospital stays for elderly patients who were suffering from ischemic heart diseases. The effect is small, but an increase of 10 percentage points in the activity-based component reduced the average length of each hospital stay by 1.28%.

**Conclusions:**

In a combined financing system such as the one prevailing in Norway, hospitals appear to respond to economic incentives, but the effect of their responses on inpatient cost is relatively meagre. Our results indicate that hospitals still need to discuss guidelines for reducing hospitalisation costs and for increasing hospital activity in terms of number of patients and efficiency.

## Background

Whether the activity-based financing of hospitals provides staff with incentives to reduce the length of each hospital stay is an empirical question that needs investigation. In this paper, we analyse how the activity-based component of a hospital’s financing system influences the average length of hospital stays (LOS) for elderly patients suffering from ischemic heart diseases in Norway.

Studies that evaluate the effects of introducing prospective financing systems that are based on the DRG system in US hospitals during the 1980s indicated a significant reduction in LOS in the range of -3 to -9% [1-10]. The results of European research are divergent, but most EU studies indicate that introducing a prospective payment system ultimately engenders negative effects on LOS, such as -24% in Hungary [11] and -4.6% in Austria [12]. All of the studies that are listed in this section have analysed the effects of fundamental system changes. In contrast, we concentrate our analysis on the effects of incremental changes in the activity-based component. The hospitals in many countries are likely to find our focus to be relevant, because many countries have implemented systems that combine activity-based financing in the DRG system with a component based on fixed payments.

Chalkley and Malcomson already delineated a theoretical understanding of changes in the financing system for non-profit hospitals [13,14], and Biørn et al. adapted this theory to the Norwegian setting [15,16]. These models often assume a trade-off between efficiency and quality in hospital production that can be shifted by various reimbursement systems. Low-powered financing systems, i.e., reimbursement systems with weak economic incentives, can give rise to serious inefficiencies in the hospital system, yet the public’s perception of the system’s healthcare services can meanwhile improve. High-powered prospective payment systems, on the other hand, increase efficiency, but can generate severe quality problems due to creaming (overtreating low-risk patients), skimping (reducing quality in various ways, such as reducing LOS), or dumping (avoiding the treatment of high-risk patients).

We have examined the level of the activity-based component over a period of 8 years, and analysed how the level correlates with LOS. To reduce the problem of intradiagnostic heterogeneity in LOS, we limit the present analysis to 3 ischemic heart diseases: angina pectoris, congestive heart failure, and myocardial infarction. We analysed our data with a log-linear regression model.

### Institutional setting

**Figure 1:**
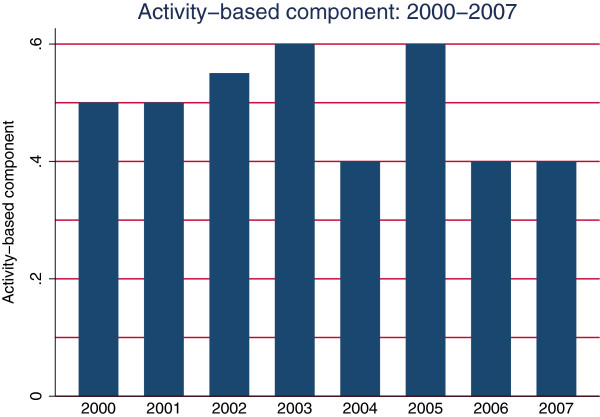
Activity-based component: 2000–2007.

The hospital sector in Norway is predominantly public with only a few non-profit, private hospitals and some for-profit hospitals that specialise in elective surgery. For a general description of the Norwegian health care system, see [17,18]. The hospital sector is organised into 4 regions that are each administered by a regional health authority. The health regions are sub-divided in geographical catchment areas that are administered by health enterprises. A health enterprise usually consists of 1–3 acute hospitals and several institutions that provide addiction therapy and psychiatric services. Each health region’s hospitals are organised hierarchically according to functions and specialties with the regional, university hospital at the top of the specialty hierarchy. Since July 1st 1997, Norwegian hospitals have had a mixed financing system consisting of a risk-adjusted capitation component and an activity-based component. The nature of the activity-based component depends on the number of patients the institution treats, the patients’ DRGs, and the national, standardised price per treatment. The activity-based component has changed several times, and constituted between 40% and 60% of expected hospital revenues in the period from 2000 to 2007 (Figure 1). The central government chooses the size of the activity-based component after holding political discussions in the parliament, so the size is exogenous to the different hospitals in Norway. Finally, Norwegian hospital physicians work on a salary basis [17].

#### Treatment of patients with heart diseases

The patients’ data were extracted from the Norwegian Patient Register. These data include all individuals in Norway who were suffering from at least one of 3 different ischemic diagnoses at the time the data were originally collected.

Angina pectoris is temporary chest pain or a sensation of pressure on the chest that occurs when the heart muscle is deprived of oxygen. It is caused by a partially narrowed artery. Its treatment is typically determined by the stability and the severity of the symptoms. When symptoms are stable and manifest mildly or moderately, the common choice of treatment is medication and modification of risk factors (e.g., smoking). When symptoms are unstable, immediate hospitalisation is usually required so that doctors can closely monitor a more intensive drug therapy and can consider the necessity of invasive procedures, such as percutaneous coronary intervention (PCI) or coronary artery bypass grafting (CABG).

Myocardial infarction is usually a medical emergency situation in which some of the heart’s blood supply is suddenly and severely reduced or cut off, causing the heart muscle (myocardium) to die because of oxygen supply deprivation. Myocardial infarction is caused by a totally blocked coronary artery, so it requires prompt intervention. In addition to drug therapy, doctors often elect to apply both PCI and CABG to patients with myocardial infarction.

Congestive heart failure is generally defined as the heart’s inability to supply sufficient blood flow to meet the body’s needs. Myocardial infarction is one of the most common causes of congestive heart failure. Treatments of congestive heart failure vary to address the various potential causes. Surgery is a valid treatment if the cause of heart failure is a narrowed or leaking heart valve or an abnormal connection between heart chambers. Blockage or severe narrowing of a coronary artery is likely to require drugs, surgery, or angioplasty. Heart transplantation may also be an option for a few otherwise healthy people who have not responded well to traditional therapy.

Both PCI and CABG treatment in Norway are centralised to specialised hospitals. PCI, which is the procedure most frequently used, is centralised to eight intervention centres. For this reason, patients, especially myocardial infarction patients who live in a catchment area with only local hospitals, may first be admitted to a local hospital and then transferred to an intervention centre outside the catchment area. In such a case, they would return to the local hospital before discharge. Since our data are hospital-based and not episode-based, the LOS for these patients might be separated into 2 or even several parts. We discuss this problem later in this paper.

## Methods

### Data

The empirical analysis is based on repeated cross-sectional data from 49 public hospitals and 4 private, non-profit hospitals. Data were delivered by the Norwegian Patient Register. The Department of Health Management and Health Economics, University of Oslo applied and received permission to use these data for this research project. The data set includes the individual patient variables of age, sex, diagnoses, DRG weights, number of comorbidities, admission type (non-elective or elective), and LOS (in days). The study also complies with the international and national ethical standards described by The National Committee for Research Ethics in the Social Sciences and the Humanities and by the Regional Committees for Medical Research Ethics (REK). The approved individual data set for our empirical data analysis is strictly confidential.

#### Defining catchment area groups

**Figure 2:**
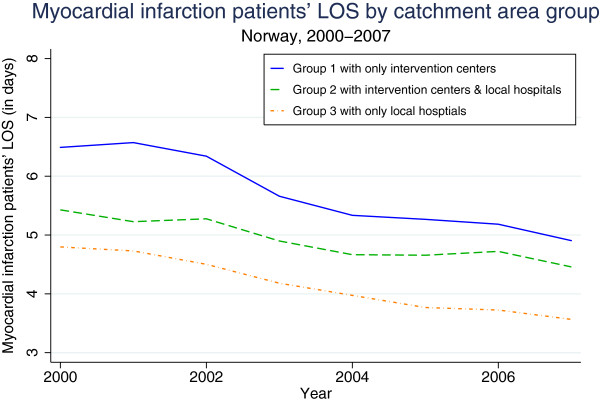
Myocardial infarction patients’ LOS by catchment area group.

As indicated, the geographic location of PCI intervention centres can influence patients’ LOS due to transfers. Unfortunately, it is not possible to identify transfers between hospitals from our data. To amend this weakness, we have defined 3 groups of hospital catchment areas according to the likelihood of such transfers. Inclusion of the group variables in the model serve as a proxy for patient transfer data. The average LOS for the different groups is shown in Figure 2. The first group (Group 1 in Figure 2) consists of catchment areas where the intervention centre is the only hospital. In this case, the patient, who is usually a patient with myocardial infarction, is likely to be fully treated within that single intervention centre. The second group (Group 2 in Figure 2) includes catchment areas with both intervention centres and local hospitals, where some of the patients might have received their entire treatment in a local hospital, some in an intervention centre, and some across transfers between an intervention centre and a local hospital. The third group (Group 3 in Figure 2) consists of catchment areas with only local hospitals that are not intervention centres. In this group, a minority of the patients were probably treated in full at the local hospital, whereas the majority (e.g., myocardial infarction patients) were more likely to be transferred between a local hospital and an intervention centre in another catchment area. As shown in Figure 2, Group 3 has the shortest average LOS due to the high transfer rate.

### Modelling the length of stay

Inspired by [12,19], we wrote our basic LOS function at the individual level as:

(1) $$y_{i}={x^{\prime }}_{i}\beta +\alpha +u_{i}$$

where *Y*_*i*_ is the logarithm of length of stay for admission number *i*, *x*^′^_*i*_ is a vector of exogenous variables, α is unobserved individual effects, *u*_*i*_ is an error term, and *β* is a vector of coefficients.

According to the literature, longer LOS is associated with older patients [20], females [12,21], patients with more comorbidities [22], patients with a higher DRG weight [22], patients with non-elective admission to the hospital [21], and the incentives of the financing system [11-13,23,24]. Furthermore, other time-related factors can influence LOS, such as technology improvements, development of outpatient care and nursing homes, and patients’ preferences for short stays [23-25]. By gleaning the literature and available data, we therefore included the following explanatory variables in the empirical analysis: age, age square, gender (female = 1, male = 0), level of ABF (the activity-based component), the DRG weight, number of comorbidities, a patient admission dummy (non-elective = 1, elective = 0), 2 dummies of ischemic heart diagnosis (myocardial infarction = 0, angina pectoris = 1, congestive heart failure = 1), 2 dummies from the 3 catchment area groups (Group 1 = 1, Group 2 = 1, Group 3 = 0), and a linear time trend variable to represent the influence of technological improvements and other time-related factors. The DRG weights and the number of comorbidities were included to account for severe cases and cases of mixed severity, which can influence resource use.

We estimated OLS regression of y on *x*_*i*_ in equation 1. In order to justify that the residuals are normally distributed, we estimated the logarithm of LOS instead of LOS itself. The coefficient *β* measures the effect of a change in regressor *x*_*i*_ on *E*(*ln*(*LOS*)|*x*). In order to investigate the possible problems of heterogeneity, we also developed a pseudo-panel model [26] by utilising Hausman-Taylor estimation [27], and we describe this model and estimation in the Appendix.

## Results

### Descriptive statistics

The original dataset includes 332,899 observations. Very few patients (1,853 or 0.6%) stayed in the hospitals for more than 30 days. Although some of these long-term stays might represent actual hospital stays, we believe that most of them are registration errors. We therefore excluded these observations from the analysis, and thus the final dataset includes 33,1046 observations. Table 1 shows the summary statistics by diagnoses and gender. The average hospital LOS is 4.4 days (column 6, Table 1). Females averaged a longer stay than did males (4.7 versus 4.3 days). The average patient age was 68.6 years, and females are about 7 years older than males (73.4 years versus 66.1 years). Thirty-eight percent of all patients that we included in the study were diagnosed with myocardial infarction, 25% with congestive heart failure, and 37% with angina pectoris. About 73% of all patients were admitted non-electively, and 10% more females than males were admitted non-electively. The patients also averaged 2 additional comorbidities, and females had more than males did (2.1 versus 1.9). However, females had a lower average DRG weight than did the males (1.2 versus 1.4).

**Table 1:** Descriptive statistics of ischemic heart diseases patients, based on the individual Norwegian patient register dataset during 2000-2007

	**Ischemic heart diseases patients**					
**Variables:**	**Myocardial infarction**	**Congestive heart failure**	**Angina pectoris**	**Male**	**Female**	**Total**
Length of stay	5.72(4.77)^1^	3.43(3.49)	3.76(3.76)	4.27(4.09)	4.70(4.48)	4.42(4.23)
Age	70.59(13.86)	66.08(11.61)	68.21(12.59)	66.05(12.51)	73.36(12.47)	68.57(12.97)
Non-elective(%)	93(0.25)	43(0.49)	73(0.44)	69(0.46)	80(0.40)	73(0.44)
No. of Comorbidities	2.20(1.76)	2.03(1.44)	1.64(1.50)	1.86(1.57)	2.12(1.66)	1.95(1.61)
DRG weight^2^	1.41(0.99)	1.49(1.65)	1.22(1.44)	1.44(1.45)	1.20(1.15)	1.36(1.36)
Myocardial infarction(%)				37(0.48)	39(0.49)	38(0.48)
Congestive heart failure(%)				28(0.45)	20(0.40)	25(0.43)
Angina pectoris(%)				36(0.48)	40(0.49)	37(0.48)
Female(%)	36(0.48)	28(0.45)	37(0.48)			34(0.48)
Observations	124449	83307	123290	216953	114093	331046

^1^Std. Dev in the parentheses.

^2^Diagnosis-related group weight (DRG) is also called cost weight for a diagnosis related group, which expresses the related resource consumption of this patient group compared to the average for all patient groups. (See the definition in the Norwegian Directorate of Health).

**Figure 3:**
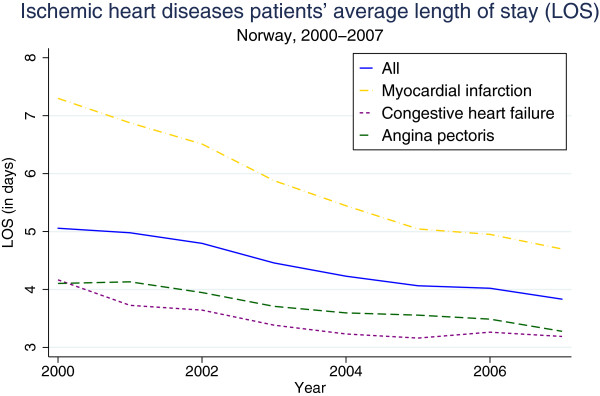
Ischemic heart diseases patients’ average length of stay (LOS).

Figure 3 shows the average LOS for all patients and then separately for the 3 diagnoses. The downward curves indicate that LOS reduced from 5.1 days in 2000 to 3.8 days in 2007 for the ischemic heart disease patient group as a whole, yet the reduction varies between the 3 groups. The reduction in LOS for myocardial infarction and angina pectoris patients is probably an effect of technological changes, in particular the introduction of PCI.

### Regression results

Table 2 displays the results from the log-linear regression at the individual level. The coefficient of the activity-based component is -0.129, which means that an increase of 10 percentage points in the activity-based component will reduce LOS by approximately 1.28% (exp(-0.129*0.1) = 98.72%). The average LOS for women was 1.9% longer than that for men. Larger effects were obtained for the health-status measures; for example, increasing the DRG weight by one unit increased LOS by 26.5%, and increasing the number of comorbidities by one prolonged LOS by 8.3%. The estimated effect of being admitted electively compared to being admitted non-electively is a 38% rise in LOS. The LOS for angina pectoris patients and congestive heart failure patients was respectively 31.3% and 32.0% shorter than the LOS for myocardial infarction patients (the reference dummy variable) after controlling for other characteristics. The effects of the catchment area groups are quite large and significant, i.e., the LOS for patients staying in catchment area group 1 and catchment area group 2 are respectively 22.8% and 1.6% longer than the LOS for patients in catchment area group 3. The coefficient of the time trend is negative and significant. The nonsignificance of age might be due to the fact that the sample was restricted to elderly people, and our choice of health-status measures might have emphasised the health effects of age.

**Table 2:** OLS regressions at the individual level

	**(1)**	**(2)**	**(3)**	**(4)**
	**All**	**Myocardial infarction**	**Congestive heart failure**	**Angina pectoris**
Age	0.001 (0.001)	-0.002 (0.001)	-0.002 (0.002)	0.014*** (0.001)
Age2/1000	0.050*** (0.007)	0.094*** (0.010)	0.077*** (0.014)	-0.074*** (0.011)
Female	0.019*** (0.003)	0.048*** (0.005)	-0.001 (0.005)	0.001 (0.004)
Activity-based component	-0.129*** (0.017)	-0.287*** (0.028)	-0.041 (0.031)	-0.039 (0.028)
DRG weight	0.265*** (0.003)	0.138*** (0.004)	0.275*** (0.004)	0.325*** (0.003)
No. of co morbidities	0.083*** (0.001)	0.102*** (0.001)	0.085*** (0.002)	0.064*** (0.002)
Non-elective	0.380*** (0.004)	0.508*** (0.009)	0.296*** (0.007)	0.515*** (0.006)
Angina pectoris^1^	-0.313*** (0.003)			
Congestive heart failure	-0.320*** (0.004)			
Group1^23^	0.228*** (0.004)	0.336*** (0.006)	0.190*** (0.010)	0.121*** (0.006)
Group2^4^	0.016*** (0.004)	0.013* (0.006)	0.042*** (0.009)	-0.012 (0.006)
Time trend	-0.065*** (0.001)	-0.100*** (0.001)	-0.039*** (0.001)	-0.039*** (0.001)
_cons	130.3*** (1.244)	201.6*** (2.082)	78.70*** (2.453)	77.66*** (1.956)
*N*	331046	124449	83307	123290
*R*^2^	0.322	0.243	0.360	0.289

Standard errors in parentheses.

*p < 0.05, ** *p* < 0.01, ***p < 0.001.

^1^Myocardial infarction is the reference dummy for both angina pectoris and congestive heart failure.

^2^Group 1 consists of catchment areas where the intervention center is the only hospital.

^3^Group 3 which consists of catchment areas with only local hospitals is the reference dummy for both Group 1 and Group 2.

^4^Group 2 consists of catchment areas with both intervention centers and local hospitals.

When we estimated the 3 diagnoses separately (shown in columns 2–4), we found that increasing the activity-based component significantly reduces LOS for myocardial infarction patients. Myocardial infarction patients also have the strongest negative time trend effects among all 3 disease types. In contrast, increasing the activity-based component has no effect on the LOS of patients with congestive heart failure and/or angina pectoris.

The pseudo-panel model in the Appendix displays results that are compatible with our individual-level analysis. This model gave far less accurate estimates, however, which is the reason why we primarily present the individual level log-linear model results. Additionally, we used a linear multilevel regression model and a negative binominal regression model. The results are similar in magnitude and significance, as Table 2 indicates.

## Discussion and conclusions

We have studied how the level of the activity-based component in the hospitals’ financing system influences the average length of hospital stays for elderly patients suffering from ischemic heart diseases. The results show that when the activity-based component increases by 10 percentage points, the average LOS of such elderly patients decreases by 1.28% or approximately 1.36 hours (1.28%*4.42 days*24 hours). In other words, the hospitals are responding to the economic incentive of an incremental change in the activity-based component, but the incentive’s ultimate impact on LOS is rather small.

We also found that other factors besides the activity-based component are associated with longer LOS, in particular older patients, females, higher DRG weight, more comorbidities, non-elective admission, and being diagnosed with myocardial infarction. These results are in accordance with findings from previous studies. The LOS for patients who had stayed in catchment area group 1 and catchment area group 2 were longer than the LOS for group 3, which verifies that the LOS for patients in catchment area group 3 are likely to have been divided due to the geographic location of the PCI centres. The time trend variable, which is assumed to capture the effects of technological progress, significantly reduces LOS. Myocardial infarction patients have the strongest negative time trend effects among the patient groups. The myocardial infarction patients are recovering faster in recent years due to the introduction of PCI treatment.

In the diagnosis-specific analyses, we discovered that increasing the activity-based component significantly reduces LOS for myocardial infarction patients but not for patients with congestive heart failure and angina pectoris. This discrepancy might come from some other factors that influence LOS among the latter 2 types of patients, such as standardisation of practice, interdisciplinary team dynamics, and physician leadership [28-32].

There has been much political debate regarding how to reduce hospital costs. The conventional view is that increasing the activity-based component results in a reduction in the length of patient stay and hence reduced costs. Our results indicate that a reduction in the activity-based component in the current high-powered prospective payment system in Norway has only a small impact on the reduction of LOS. Therefore, an incremental change in the activity-based component does not appear to give hospitals strong enough incentives to reduce inpatient costs considerably for this specific patient group. The small effect observed in this analysis might also have been caused by some hospitalisation regulations and instructions for the treatment of these 3 ischemic heart diseases. For instance, these patients must stay at the hospitals for a fixed amount of days to fulfil the standard treatment plans for these diseases. On the other hand, our results indicate that a higher activity-based component is still generally recommendable, because it has a positive effect on hospital activities in terms of number of patients and hospital efficiencies [13,14,24]. Another factor is that Norwegian physicians work on a salary basis and do not receive pay according to the number of treated patients. They might not have the right economic incentives to work directly to reduce the length of stay.

Our scope of interest in this paper has been restricted to the effect of the activity-based component on LOS for elderly patients suffering from heart diseases. One limitation of the present study is that we focus only on patients with these 3 ischemic heart diseases. Due to the treatment specialties for this patient group, the effects on LOS probably differ for other patient groups, especially for diagnoses with no prevailing treatment instructions. The other limitation of this study is that we did not account for other factors that might influence LOS while conducting our analyses, such as waiting time, supply of beds, staffing ratios, patients’ rate of recovery, hospital and physician characteristics [26,33], and specific indicators that reflect the severity of illness for this patient group, e.g., the number of affected vessels and the ejection fraction. It would be interesting to explore how these factors impact LOS, as well. However, it is reasonable to assume that there is no correlation between these factors and the activity-based component, so we expect none of these factors to influence the activity-based component’s effect on LOS.

## Appendix

If the unobserved individual effects *α* are uncorrelated with the explanatory variables in *x*′_*i*_ in equation 1, we can get the consistent and unbiased estimator by simply using OLS in the repeated cross-sectional dataset. However, in reality, the unobserved individual effects are likely to be correlated with some or all of the explanatory variables, which creates the unobserved heterogeneity problem. For example, one patient might have more comorbidities than other patients due to the severity of his or her diseases, such as the number of affected vessels, ejection fraction, etc. Therefore, OLS might lead to inconsistent estimators. If we have genuine panel data, then we can easily solve this problem by using the fixed effects model or the Hausman-Taylor (HT) model [27] in equation 1. Unfortunately, we had access to only repeated cross-section data, so we could not follow the same individuals over time. This limitation implies that the traditional panel approach cannot be used directly in equation 1.

Deaton [26] suggested using the pseudo-panel technique to perform panel estimation at the aggregated level. We first had to group the individuals into pseudo-cohorts based on a fixed membership that remained the same throughout the entire period of observation. Here, we defined each cohort C for every combination of single birth year and gender. In order to make the cohort sizes fairly equal across cohort groups, we merged the oldest and youngest birth year for male and female patients separately (birth year < =1911 or > =1967 for males and birth year < =1908 or > =1958 for females). Hence, each individual patient in the data set belongs to exactly one cohort. This new data set is a pseudo-panel or a synthetic panel with repeated observations over T periods and C cohorts. It should also be noted that we reduced the number of observations from 331,046 to 847 with this cohort grouping, indicating that the estimates from the pseudo-panel method are less biased but suffer from less precision.

The aggregation of all observations to the cohort level results in:

(2) $${\bar{y}}_{\mathrm{ct}}={\bar{x^{\prime }}}_{\mathrm{ct}}\beta +{\bar{\alpha }}_{c}+{\bar{u}}_{\mathrm{ct}}$$

where ${\bar{y}}_{\mathrm{ct}}$ are the averages of all observed *ln*(*LOS*)’s in cohort *c* at time period *t*, and similarly for the other variables in the model. ${\bar{\alpha }}_{c}$ is the fixed cohort effects, which is correlated with ${\bar{x\prime }}_{\mathrm{ct}}$ (we assume ${\bar{\alpha }}_{\mathrm{ct}}={\bar{\alpha }}_{c}$ in equation 2). The dependent and the explanatory variables in equation 2 are weighted with the square root of the cohort size before estimating, because the sample cohort means should be the more reliable proxies for the population cohort means when the corresponding cohort is large [26]. We can then estimate equation 2 with the fixed effects model, and this model gives the within estimator [26-31]. Even if the within estimator is a consistent and unbiased estimator, it suffers from 2 significant defects [34]. First, all time-invariant variables are eliminated by the data transformation, so their coefficients cannot be estimated (for example, the coefficient of female is removed). Second, the within estimator is not fully efficient, because it ignores the variation across individuals.

An IV estimator with neither of these defects was proposed by [27]. The HT estimator exploits the assumption that the explanatory variables are uncorrelated with the fixed cohort effects. The HT model [27] can be written as:

(3) $${\bar{y}}_{\mathrm{ct}}={\bar{x\prime }}_{1\mathrm{ct}}\beta_{1}+{\bar{x\prime }}_{2\mathrm{ct}}\beta_{2}+{\bar{z\prime }}_{1c}\gamma_{1}+{\bar{z\prime }}_{2c}\gamma_{2}+{\bar{\alpha }}_{c}+{\bar{u}}_{\mathrm{ct}}$$

Where ${\bar{x\prime }}_{1\mathrm{ct}}$ and ${\bar{x\prime }}_{2\mathrm{ct}}$ are vectors of time-varying variables, and ${\bar{z\prime }}_{1c}$ and ${\bar{z\prime }}_{2c}$ are vectors of time-invariant variables. In addition, ${\bar{x\prime }}_{1\mathrm{ct}}$ and ${\bar{z\prime }}_{1c}$ are 2 vectors of exogenous variables in that they do not correlate with ${\bar{\alpha }}_{c}$ and ${\bar{u}}_{\mathrm{ct}}$, while ${\bar{x\prime }}_{2\mathrm{ct}}$ and ${\bar{z\prime }}_{2c}$ are 2 endogenous variables that correlate with ${\bar{\alpha }}_{c}$ but not with ${\bar{u}}_{\mathrm{ct}}$. Because ${\bar{x\prime }}_{2\mathrm{ct}}$ and ${\bar{z\prime }}_{2c}$ correlate with ${\bar{\alpha }}_{c}$, estimating equation 3 by using the random effects model does not result in consistent parameters. If we estimate equation 3 by using the fixed effects model, we are not estimating *γ*_1_ or *γ*_2_, because this model eliminates ${\bar{z\prime }}_{1c}$ and ${\bar{z\prime }}_{2c}$ .

After using the Hausman specification test [33], we chose the exogenous variables of ${\bar{x\prime }}_{1\mathrm{ct}}=\left(\mathrm{ABF},\mathrm{Group}1,\mathrm{Group}2,\mathrm{Year}\right),{\bar{z}}_{1c}=\left(\mathrm{Female}\right)$,to implement the HT estimator. In other words, we allowed some regressors (i.e., age, comorbidities, non-elective, DRG weight, angina pectoris, and congestive heart failure) to correlate with the unobserved individual-level random effect. The estimation results are presented in Table 3.

**Table 3:** Hausman-Taylor estimation at the cohort level

	**HT**
Age2/1000	0.150*** (0.019)
Female	-0.046 (0.069)
Activity-based component	-0.128*** (0.026)
DRG weight	0.308*** (0.019)
No. of comorbidities	0.068*** (0.014)
Non-elective	0.345*** (0.065)
Angina pectoris^1^	-0.083 (0.051)
Congestive heart failure	-0.266*** (0.067)
Group1^23^	0.061 (0.068)
Group2^4^	-0.022 (0.057)
Time trend	-0.069*** (0.003)
_cons	137.5*** (5.511)
*N*	864
Spec. Test^56^	$\chi_{9}^{2}=18.91$

Standard errors in parentheses.

*p < 0.05, **p < 0.01, ***p < 0.001.

^1^Myocardial infarction is the reference dummy for both angina pectoris and congestive heart failure.

^2^Group 1 consists of catchment areas where the intervention center is the only hospital.

^3^Group 3 which consists of catchment areas with only local hospitals is the reference dummy for both Group 1 and Group 2.

^4^Group 2 consists of catchment areas with both intervention centers and local hospitals.

^5^We choose the exogenous variables X1 = (ABF, Group1, Group2, Year), Z1 = (Female) to implement the HT estimator.

^6^Hausman’s test based on the difference between the within and HT estimator gives an observed $x_{9}^{2}=18.91$. Compared to the Hausman’s test for fixed and random effects which gives $x_{10}^{2}=89.98$, we can see that HT approach does improve the efficiency in the fixed effects model.

Moreover, when we compared log-linear regression at the individual level (Table 2) with HT estimation at the cohort level (Table 3), we revealed that the sign and the size of the explanatory variables are similar except for gender, angina pectoris, and 2 catchment area group variables (group 1 and group 2). The 4 variables now turn out to be insignificant, which reflects a trade-off between estimate accuracy and estimate bias. The results at the cohort level are less biased, but they lack the prize of precision. As one can see by looking at Table 3, the confidence intervals of the HT estimates are within the confidence intervals of the log-linear estimates, and the standard errors at the cohort level are larger than they are at the individual level. This result is normal, because the number of cohort groups is lower than the number of individual observations. Therefore, we do not analyse the data separately with 3 different diagnoses at the cohort level in order to prevent the standard errors that are largely spread out at the cohort level. Our final results are based on a log-linear regression at the individual level (Table 2), because it is more precise.

## Competing interests

The authors declare that they have no competing interests.

## Authors’ contributions

JY participated in the design of the study, assisted with the analysis and interpretation of the data, and drafted the manuscript. HL, TH, and FD contributed to the design of the study, helped to interpret the data, and were involved in critically revising the manuscript. All authors read and approved the final manuscript.

## Pre-publication history

The pre-publication history for this paper can be accessed here:

http://www.biomedcentral.com/1472-6963/13/172/prepub
